# Preparation and Characterization of Nanocomposite Films Containing Nano-Aluminum Nitride and Cellulose Nanofibrils

**DOI:** 10.3390/nano9081121

**Published:** 2019-08-03

**Authors:** Shuangxi Nie, Yuehua Zhang, Linmao Wang, Qin Wu, Shuangfei Wang

**Affiliations:** 1 State Key Laboratory of Biobased Material and Green Papermaking, Qilu University of Technology, Shandong Academy of Sciences, Jinan 250353, China; 2 School of Light Industry and Food Engineering, Guangxi University, Nanning 530004, China

**Keywords:** bagasse pulp, cellulose nanofibrils, cellulose nanocomposites, nano-aluminum nitride

## Abstract

Nanocomposites consisting of cellulose nanofibrils (CNFs) and nano-aluminum nitride (AlN) were prepared using a simple vacuum-assisted filtration process. Bleached sugarcane bagasse pulp was treated with potassium hydroxide and sodium chlorite, and was subsequently ultra-finely ground and homogenized to obtain CNFs. Film nanocomposites were prepared by mixing CNFs with various AlN amounts (0–20 wt.%). X-ray diffraction revealed that the crystal form of CNF-AlN nanocomposites was different to those of pure CNFs and AlN. The mechanical performance and thermal stability of the CNF-AlN nanocomposites were evaluated through mechanical tests and thermogravimetric analysis, respectively. The results showed that the CNF-AlN nanocomposites exhibited excellent mechanical and thermal stability, and represented a green renewable substrate material. This type of nanocomposite could present great potential for replacing traditional polymer substrates, and could provide creative opportunities for designing and fabricating high-performance portable electronics in the near future.

## 1. Introduction

Nowadays, electronic devices are developing rapidly, since manufacturers are aiming at achieving miniaturization, high power, etc. [1,2]. The partial overheating of electronic devices raises inevitable concerns of thermal failure, performance degradation, and shortened life span. Controlling the heat dissipated by electronic devices has become an increasingly urgent problem [3]. Developing materials with high thermal conductivity, low thermal expansion coefficient, low dielectric constant, high resistivity, and low cost has become an urgent challenge when manufacturing electronic devices [4,5]. Polymer materials have attracted increasing attention due to their excellent processability and low costs [6,7]. Cellulose is the most abundant and renewable natural polymer on Earth and is also inexpensive and biodegradable [8,9,10]. Thus, the development and utilization of cellulose represents a research hot spot [11,12,13]. Cellulose nanofibrils (CNFs) have large specific surface areas, low density (1.6 g/cm^3^), and a large number of –OH side groups, which help them graft onto chemicals to obtain different surface properties [14,15,16,17]. Since they are able to form hydrogen bonds, CNF macromolecules are more tightly packed than cellulose macromolecules, and sheet materials prepared using CNFs exhibit higher physical strength [18,19].

At present, the thermal conductive materials used to manufacture electronic products mainly consist of isotropic polymer composites and thermal conductive fillers because of their good processing performance, good flexibility, and low cost [20]. Compared to isotropic heat conducting materials, CNF films exhibiting anisotropic thermal conductivity and ideal mechanical properties can dissipate heat in the direction of the plane. This observation is of great significance for developing the next generation of portable and foldable electronic devices [21]. However, CNFs have limitations as a thermal conductive material. One way to enhance the thermal conductivity of CNFs is to introduce a high thermal conductivity filler, such as alumina, aluminum nitride (AlN), boron nitride, and silicon nitride, into the CNF matrix [22,23,24]. AlN particles have been widely studied because of their high thermal conductivity (320 W/mK), high resistivity (greater than 1014 Ω), low dielectric constant and dielectric loss, and lack of toxicity. Moreover, AlN is an ideal material for applications requiring materials exhibiting both good thermal conductivity and electrical insulating properties [25]. Thus, AlN thin films are often used for mechanical, electrical, and optical applications [26]. The preparation of CNF-AlN composites can provide new ideas for solving the key poor thermal conductivity concern of cellulosic materials [27]. However, the polarity and hydrophilicity of CNFs are very strong, while AlN is non-polar and hydrophobic; therefore, they present significantly different polarities. Furthermore, the water dispersion of such composites is not good.

In this study, we prepared a CNF-AlN nanocomposite using a simple vacuum-assisted filtration process. Bleached sugarcane bagasse pulp was treated with potassium hydroxide and sodium chlorite, and was subsequently ultra-finely ground and homogenized to obtain CNFs. Film nanocomposites were prepared by mixing CNFs and AlN after ultrasonication. The thermal stability of the CNF-AlN nanocomposites was evaluated. This type of nanocomposites presents great potential for replacing the traditional polymer substrates, and provides creative opportunities for designing and fabricating high-performance portable electronics in the near future.

## 2. Materials and Methods

### 2.1. Raw Materials and Chemicals

Bleached bagasse pulp was purchased from Guangxi Guitang Co., Ltd. (Guangxi, China). The raw material was obtained through caustic soda pulping, using an alkali content of 17.5%, liquid ratio of 1:3.5, heating time of 45 min, maximum cooking temperature of 165 °C, and holding time of 110 min. The bleaching of bagasse fibers was carried out using the CEPP_1_P_2_ process. The whiteness of the bleached bagasse pulp was approximately 85% ISO. Potassium hydroxide and sodium chlorite were purchased from Aladdin (Shanghai, China), aluminum nitride (nanopowder, <100 nm particle size) was purchased from Sigma-Aldrich (Saint Louis, MO, USA), and glacial acetic acid was purchased from Tianjin Zhiyuan Chemical Reagent Co., Ltd. (Tianjin, China). All reagents were of medicinal or analytical quality.

### 2.2. Experimental Methods

#### 2.2.1. Preparation of CNFs

The concentration of purified pulp was adjusted to 3 wt.%, and then ultrafine grinding was carried out using an ultrafine grinder (MKZA 10-15JIV, Kawaguchi, Japan). The speed of the millstone was set to 1500 rpm, the gap of the mill disc was adjusted to −100 μm, and the pulp underwent six grinding 6 cycles. The samples were homogenized using a high-pressure homogenizer (M-110EH-30, Westwood, CA, USA). When the pressure reached 350 bar, the samples passed through the homogenizer five times, while at 1500 bar, the samples passed through the homogenizer 15 times. After the high-pressure homogenization process, uniform CNFs were obtained.

#### 2.2.2. Preparation of CNF-AlN Composite Films

The CNF suspension was diluted to a concentration of 0.1 wt.%, and then AlN was added to obtain a CNF-AlN mixed suspension. The amounts of added AlN were 0, 2, 5, 10 and 20 wt.% of the CNF amount. The mixed suspension was ultrasonically treated for 4 h with a power of 800 W. Then, a magnetic stirrer was used to stir for 6 h at a 400 rpm speed. The mixed suspension was vacuum filtered through a polytetrafluoroethylene (PTFE) membrane (pore size of 0.22 μm, diameter of 9 cm). The PTFE filter and wet CNF samples were both transferred to a sheet former (HAAGE Sheet Former BB, Mülheim an der Ruhr, Germany), where they were dried for 50 min at 50 °C.

### 2.3. Analysis Methods

#### 2.3.1. Transmission Electron Microscopy (TEM) Analysis

We diluted the CNFs and AlN to a concentration of 0.008 wt.% and then the sample was dripped onto the supporting film and dried. Furthermore, 1% uranium acetate was used to stain the film for 20 min in the dark and then the excess dye was absorbed using the filter paper and then dried naturally. Lastly, TEM (HT7700, Tokyo, Japan) was used to analyze the samples.

#### 2.3.2. Scanning Electron Microscopy (SEM) Analysis

A small piece of CNF-AlN composite film was fixed onto the loading stage of a SEM device using conductive adhesive. The surface was sprayed using a gold spraying current of 10 mA. The cross-section detection of membrane is to fix the membrane using liquid nitrogen and then conductive adhesive was used to fix it onto the loading stage. The cross section was sprayed with gold using an injection current of 10 mA. The surface and cross-sectional morphologies of the samples were analyzed using SEM (Phenom F16502, Eindhoven, Netherlands).

#### 2.3.3. Ultraviolet-Visible (UV-Vis) Spectrum Analysis

The transmittance of the CNF-AlN composite films was analyzed using an ultraviolet-visible (UV-Vis) spectrophotometer (Lambda 950, Waltham, MA, USA). First, air was used as reference, and the composite film was cut into 0.9 × 4 cm strips, which were placed in a petri dish. The measurement range was 190–1100 nm and the measuring speed was 50 nm/s.

#### 2.3.4. Fourier-Transform Infrared Spectroscopy (FTIR) Analysis

The CNF-AlN composites were analyzed using FTIR (TENSOR II, Ettlingen, Germany). First, the CNF-AlN mixture was freeze-dried to convert it into powder and potassium bromide (KBr) was added into the oven, where the powder was dried for 4 h. During the test, we first used the KBr lapping press as background, then mixed the sample with KBr at a 1:100 ratio. The pressing pressure and time were 50–60 MPa and 2 min, respectively. The resolution of the instrument was 0.4 cm^−1^ and the test was performed in the 400–4000 cm^−1^ range.

#### 2.3.5. X-Ray Diffraction (XRD) Analysis

The CNF-AlN composite thin films were cut into 1.5 × 1.5 cm pieces on the carrier platform and their degree of crystallinity was analyzed by using high resolution XRD (MINFLEX600, Tokyo, Japan). The test parameters were as follows: Cu Kα radiation, the wavelength of 0.15418 nm, scanning range of 10°–70°, the scanning rate of 5°/min, tube voltage of 40 kV, and tube current of 30 mA.

#### 2.3.6. Mechanical Properties Analysis

The CNF-AlN composite film was cut into 5 × 50 mm rectangles. The mechanical properties of the samples were tested using a vertical universal tension machine (AMETEK LS1, Berwyn, PA, USA). The test conditions were as follows: The distance of the sample was 2 cm, the tensile speed was 1 mm/min, and five samples were tested in parallel.

#### 2.3.7. Thermogravimetric (TG) Analysis

Thermogravimetric analysis of the freeze-dried samples was carried out using a synchronous thermal analyzer (STA 449F5, Selbu, Germany). The experimental conditions were as follows: samples weighing approximately 10 mg were collected as the temperature increased from 25 to 800 °C under nitrogen protection, the scanning rate was 10 °C/min, and the nitrogen flow rate was 20 mL/min.

## 3. Results and Discussion

### 3.1. Characterization of CNFs and AlN Suspension

**Figure 1:**
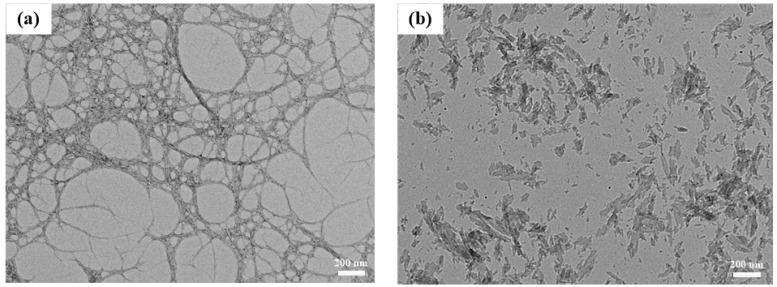
TEM images of (**a**) cellulose nanofibrils (CNFs) and (**b**) nano-aluminum nitride (AlN).

**Figure 2:**
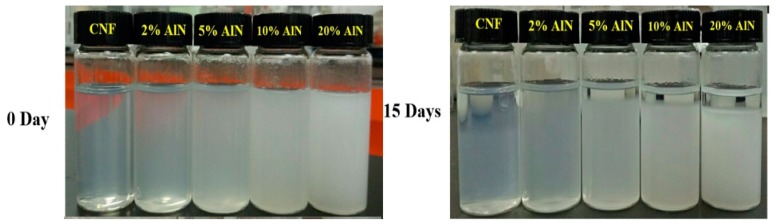
CNF-AlN suspensions with different contents.

Due to the complex multilayer structure of plant fibers and the high number of hydrogen bonds, the mechanically prepared CNFs presented a wide diameter distribution [28]. For fiber composites, the aspect ratio of the fiber affects its ability to combine with other materials, which largely determines the mechanical properties of the composite [29,30]. Figure 1 illustrates the TEM images of CNFs and AlN. The convergence of CNFs and single root CNFs can be observed from Figure 1a. The width of the CNFs prepared using a mechanical method was smaller than 100 nm, the length and width were relatively large, and the fibers were intertwined with each other into a tree-like structure. Such structures could explain the relatively superior mechanical and thermal properties of CNF composites. The width of AlN in Figure 1b was also within 100 nm, which allowed AlN to be better dispersed in CNFs, thus being beneficial to the preparation of CNF-AlN composites with superior performance. The mixing uniformity of CNFs and AlN greatly influenced the optical and mechanical properties of the prepared composite films. The CNF-AlN mixed suspension was sonicated, and the suspension was analyzed. Subsequently, the suspension was allowed to stand for 15 days while it dispersed, as shown in Figure 2. It can be seen that pure CNFs were dispersed in the aqueous solution to form a transparent CNF suspension.

**Figure 3:**
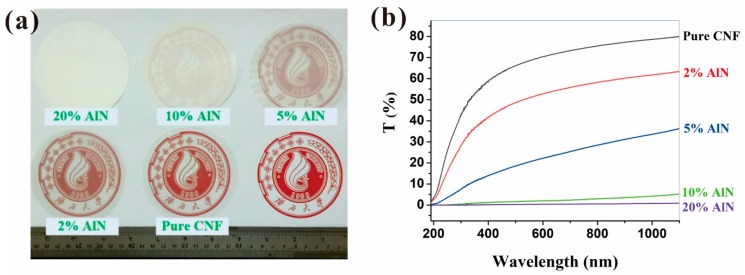
Optical properties of CNF-AlN composites: (**a**) Optical images and (**b**) transmittance.

To examine the optical properties of the CNF-AlN composite films, the prepared CNF-AlN composite films were photographed in a well-lit laboratory and the changes in their appearance were analyzed. The results are shown in Figure 3. As can be seen from Figure 3a, when the composite film was placed on the red Guangxi University logo as background, the pure CNF film allowed the logo to be clearly visible in the background. As the amount of AlN added to CNF increased, the transparency of the composite film gradually decreased. When the AlN content was 20%, the logo was no longer visible. To reduce the influence of the human factor on the experimental results, changes in the light transmittance of CNF-AlN composites containing different amounts of AlN were measured using an UV-vis spectrophotometer. The results are shown in Figure 3b. The transmittances of CNF-AlN composites containing different amounts of AlN were quite different. The transmittance of the pure CNF films could reach approximately 80%. The transmittance of the CNF-AlN composite containing 5% AlN was reduced to 35%. When the amount of added AlN exceeded 10%, the light transmittance of the composite material was already reduced to 5% or less. Paper with custom optical properties could be used for various purposes, and pure CNF films exhibiting good light transmission could be used for electronic displays, and a composite paper containing 20 wt.% AlN could be used as opaque substrate in flexible solar panels.

### 3.2. Microscopic Analysis of CNF-AlN Composites

**Figure 4:**
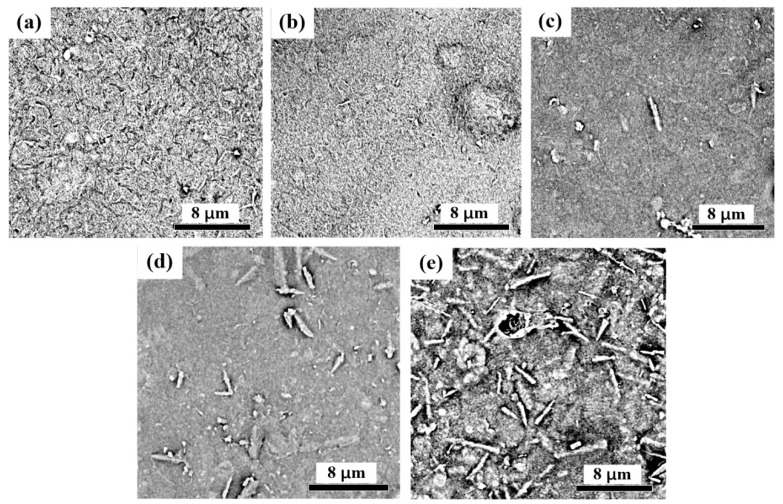
SEM images of the surfaces of CNF-AlN composites: (**a**) Pure CNFs; CNFs containing (**b**) 2% AlN, (**c**) 5% AlN, (**d**) 10% AlN, and (**e**) 20% AlN.

**Figure 5:**
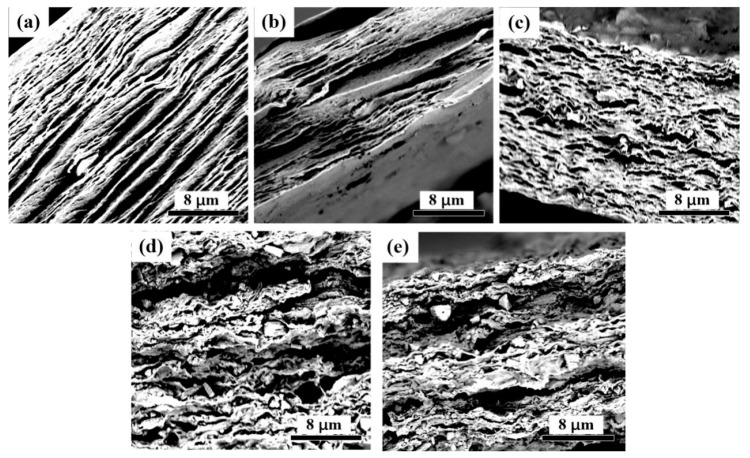
SEM images of the cross section of CNF-AlN composites. (**a**) Pure CNFs and CNFs containing (**b**) 2% AlN, (**c**) 5% AlN, (**d**) 10% AlN, and (**e**) 20% AlN.

The surface and cross section of the CNF-AlN composite were observed by SEM, as shown in Figure 4 and Figure 5. After the image in Figure 4 was magnified 10,000 times, we were able to observe that that AlN was uniformly dispersed in the CNF matrix. As the amount of added AlN increased, the AlN distribution is highly uniform and that AlN is interlaced with CNFs in a network structure. This was due to the interweaving between the CNF macromolecules to form a network-like porous sheet material. As a result, the surface of the film in Figure 4a presented cracks. As AlN was added to CNFs, the interfiber spaces were filled (Figure 4b,c). However, as the amount of added AlN increased, too much AlN would become exposed at the surface (Figure 4d,e).

Figure 5 shows a cross-sectional topography of the composite film. Since the hydrogen bonds between the fibers caused the fibers to form a complex multilayer structure, the CNF film formed a distinct layered structure (Figure 5a). After AlN was added, the AlN particles became dispersed in the cellulose matrix, and when the amount of added AlN was 5% or smaller, the layered structure was still conspicuous (Figure 5b,c). However, as the amount of added AlN increased, the layered structure of the composite material gradually became blurred (Figure 5d,e). When the amount of added AlN was 10% or more, a large amount of AlN in the CNFs became agglomerated and destroyed the layered structure of CNFs. Destroying the layered structure of the composite would lead to a reduction of the mechanical properties of the composite, while further hindering the contact between AlN particles, thus preventing them from forming a network structure.

### 3.3. FTIR Analysis of CNF-AlN Composites

**Figure 6:**
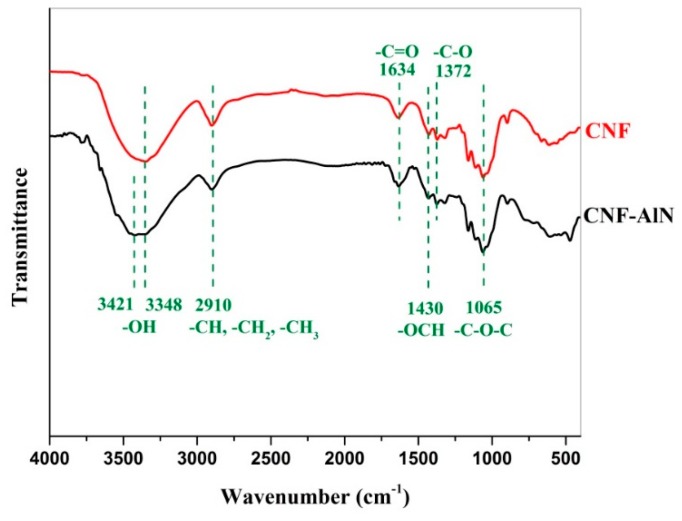
FTIR spectra of CNF and CNF-AlN composites.

The functional groups of CNF-AlN composites were analyzed using FTIR to study the effect of the chemical structure on the properties of composites. We performed FTIR analysis on pure CNFs and CNF composites containing 10% AlN. The obtained spectra are shown in Figure 6. It can be seen from Figure 6 that the spectrum of the CNF composite containing 10% AlN was very similar to that of pure CNFs, where the 3200–3500 cm^−1^ peak represented a hydrogen-bonded –OH stretching vibration peak, and the 2910 cm^−1^ peak represented the symmetric stretching vibration absorption peak of C–H or –CH_2_. The 1430 cm^−1^ peak corresponded to the –OCH in-plane bending vibration, and the 1372 cm^−1^ and 1065 cm^−1^ peaks were attributed to the stretching vibrations of the C–O asymmetric bond and the C–O–C bond, respectively [31,32]. Pure AlN exhibited a very strong absorption peak at 731 cm^−1^, which represented the stretching vibration absorption peak of Al–N. However, this absorption peak was not detected in the spectrum of the CNF-AlN composite. Since AlN has a melting point of roughly 2000 °C it will not easily dissociate. Moreover, the entire material preparation process used only ultrasonic and vacuum filtration treatments, the two treatment methods would not have been sufficient to break the Al–N bonds. It is most likely that the phonon (lattice vibration) is not observed because the cross section is low or maybe, due to the low transparency (as shown in Figure 3), the light cannot easily penetrate the sample.

### 3.4. XRD Analysis of CNF-AlN Composites

**Figure 7:**
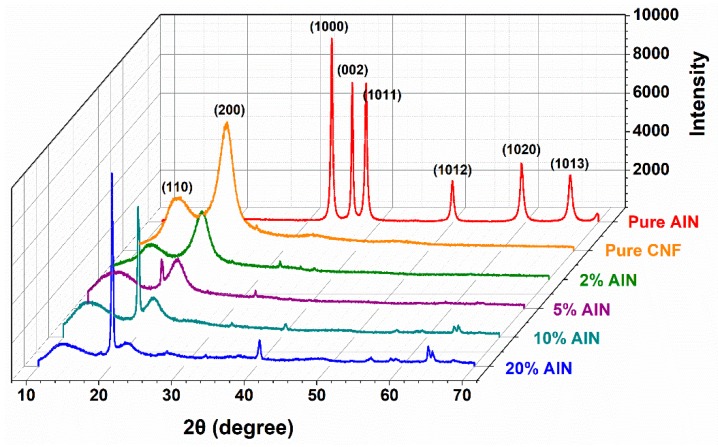
XRD spectra of the pure AlN, pure CNF and CNF-AlN composites.

Analyzing XRD patterns is an effective method to determine the crystallinity of a sample. Crystallinity is an important parameter for characterizing the properties of polymers. Some physical and mechanical properties of polymers are closely related to their crystallinity. Figure 7 shows the X-ray diffraction pattern of the pure AlN, pure CNF and AlN/CNF samples. It can be seen that the XRD patters of pure CNF displayed peaks at 2θ = 16.68°, and 22.1°, corresponding to the (110) and (200) crystallographic planes, which are in agreement with the characteristic diffraction peaks of cellulose Iβ [33]. The diffraction peaks of AlN mostly concentrate in the range of 2θ = 36°–70°. As the amount of added AlN increased, the intensities of the diffraction peaks of the composites at the (110) and (200) planes gradually decreased. When the AlN content of the composite was 10%, additional characteristic diffraction peaks of AlN could be observed at 2θ of 39° and 63.6° [26]. Moreover, as the AlN content increased, the intensity of the characteristic diffraction peak of AlN gradually increased.

### 3.5. Mechanical Properties of CNF-AlN Composites

**Figure 8:**
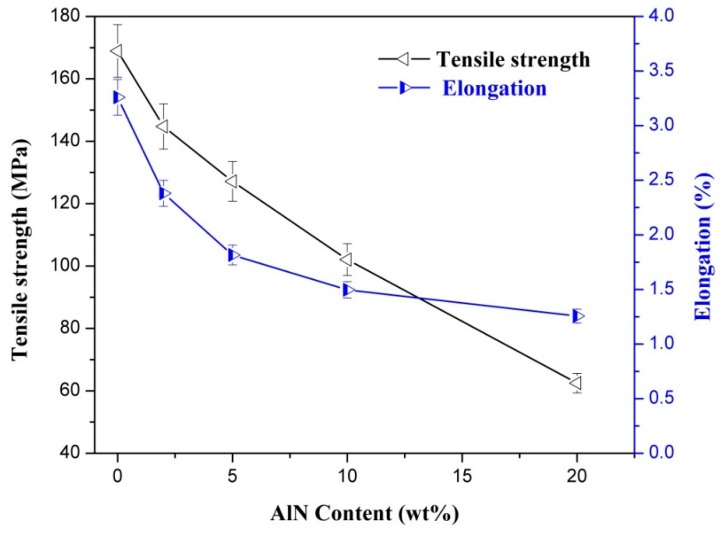
Mechanical properties of CNF-AlN composite reinforced with different amounts of AlN: (a) Tensile strength; (b) elongation.

Mechanical properties are very important performance indices of CNF-AlN composites, which play decisive roles in their application ranges. Figure 8 shows the mechanical properties testing results of CNF-AlN composites. It can be seen from Figure 8 that the tensile strength and elongation at break of the CNF-AlN composite could reach 142.5 MPa and 0.24%, respectively, when the added AlN amount was 2%. This occurred because when the amount of added AlN was small, CNF acted like an adhesive to tightly bind the AlN nanosheets together. Thus, the AlN nanosheets in the CNF became well aligned and the AlN particles were sufficiently connected with each other as well as with the CNF molecules. At the same time, a large number of amino (-NH_2_) and hydroxyl (–OH) groups existed at the edge of AlN, and these easily formed hydrogen bonds with the –OH and carboxyl (–COOH) groups on the surface of CNF [20]. A nanocomposite exhibiting high mechanical strength would be formed by hydrogen bonding. It can be seen from the cross-sectional morphology of the CNF-AlN composite film (Figure 5) that the uniform CNF-AlN suspension formed a three-dimensional structure presenting stacked layers and compact, dense, and regular features when vacuum filtration and high vacuum conditions were used. An orderly and uniform structure was formed between CNF and AlN through strong interfacial interactions. This special structure also represented an important reason for the formation of nanocomposites exhibiting high mechanical strength. As the amount of added AlN increased, the tensile strength and elongation at break of the composites were gradually reduced, as AlN itself exhibited high brittleness and was not easily interwoven into a film. For the CNF-AlN composites, as the amount of added AlN increased, gaps occurred between the CNF fibers and a large amount of AlN was agglomerated between the CNF fibers, destroying the layered structure of the composite. The strength of the hydrogen bonds was inversely related to the AlN content, resulting in a gradual decrease in tensile strength and elongation at break of the composite.

### 3.6. Thermal Stability of CNF-AlN Composites

**Figure 9:**
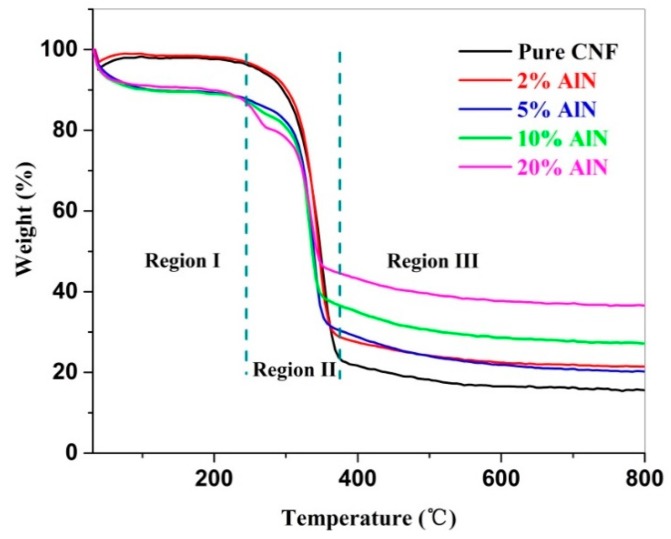
Thermogravimetric (TG) curves of pure CNFs and CNF-AlN composites.

In recent years, CNF composites have been increasingly used and are expected to substitute traditional organic polymer materials. However, they also undergo thermal degradation at high temperatures. Recently, scholars have focused on studying the thermal stability of cellulose [34,35,36,37]. These studies could provide a clearer understanding of the pyrolysis mechanism of cellulose and provide references for obtaining CNF composites with superior thermal properties. The TG curves of the CNF-AlN composite containing different AlN amounts obtained at a heating rate of 10 °C/min are shown in Figure 9. The TG curves of each sample could be divided into three regions based on the changes in weight percentage of the sample as the temperature increased. As can be seen from Figure 9, the 25–220 °C range represented Region I, defined as the initial mass loss phase, and the CNF-AlN composite materials containing different amounts of AlN presented large mass losses in this region. The mass loss in Region I was mainly due to the evaporation of water from the CNF-AlN composites, as no thermal degradation occurred at this stage [14]. The final temperature of Region I (220 °C) was defined as the thermal degradation onset temperature of the sample. Region II of the TG curves of the samples represented the main mass loss phase. As shown in Table 1, the residual mass of the pure CNF sample at the end of Region II was 25.5%, while the residual mass of the CNF-AlN composite at the end of Region II was above 32%. This indicated that adding AlN to CNFs could reduce the thermal degradation of CNF composites in Region II. After Region II, the mass loss rate of the CNF-AlN composites decreased. These areas of the TG curves were defined as Region III and extended from this point to the final test temperature of 800 °C. For Region III, when the temperature exceeded 500 °C, cellulose would decompose to produce a variety of low molecular weight products [38]. At the final test temperature of 800 °C, the final residues of pure CNFs and CNF-AlN composites containing 2%, 5%, 10%, and 20% AlN were 15.4% ± 0.4%, 21.4% ± 0.2%, 20.0% ± 0.6%, 27.0% ± 0.2%, and 36.6% ± 0.4%, respectively (Table 1). It can be seen that as the AlN content increased, the mass loss of the CNF-AlN nanocomposites decreased, while the thermal stability of the CNF-AlN nanocomposites increased. Therefore, the AlN in the composite could better dissipate heat and limit the movement of the CNF polymer chains, causing the CNF polymer chains to begin to degrade at higher temperatures [39].

## 4. Conclusions

In this study, we prepared a CNF-AlN nanocomposite via a simple vacuum-assisted filtration process. Film nanocomposites were prepared by mixing CNFs and AlN after ultrasonication. We obtained CNF-AlN nanocomposites with excellent mechanical flexibility and thermal stability. This type of nanocomposites exhibits a wide-range of applications. Pure CNF films presenting good transmittance could be used for electronic displays, and the poor transmittance films could be useful for energy storage products.

## Figures and Tables

**Table 1:** Thermal stability of CNF-AlN composites.

Sample	Mass Residue (wt.%)		
	Region I	Region II	Region III
**Pure CNFs**	97.6 ± 0.2	25.5 ± 0.5	15.4 ± 0.4
**CNF-2%AlN**	98.3 ± 0.1	32.8 ± 0.3	21.4 ± 0.2
**CNF-5%AlN**	89.5 ± 0.1	33.5 ± 0.8	20.0 ± 0.6
**CNF-10%AlN**	89.4 ± 0.4	39.9 ± 0.2	27.0 ± 0.2
**CNF-20%AlN**	90.5 ± 0.4	47.5 ± 0.7	36.6 ± 0.4
